# Supplementary material for: The Association between Nutrition, Physical Activity, and Cardiometabolic Health at 6 Months following a Hypertensive Pregnancy: A BP2 Sub-Study
Source: Nutrients. 2023 Jul 25;15(15):3294. doi: 10.3390/nu15153294 (PMC10421276; doi:10.3390/nu15153294)
Supplement: Supplementary file 1 [file nutrients-15-03294-s001.zip › nutrients-2505083-supplementary.pdf]

Supplementary Materials

Table S1. BP^2's three arms of postpartum intervention [1].

(1)	**Optimised usual care**	Standard postpartum management for HDP:<br>• Information packages provided to study participants and their primary healthcare provider.<br>• Participants attended a follow-up visit at 6-months postpartum with their primary healthcare provider.
(2)	**Brief education intervention**	Participants attended a dedicated postpartum clinic at 6 months postpartum for measurements as well as:<br>• CVD risk assessment and lifestyle education provided through a consultation with a physician and dietitian.<br>• Provision of information resources regarding lifestyle behaviour changes.
(3)	**Extended lifestyle intervention**	Participants attended a dedicated postpartum clinic at 6 months postpartum for measurements as well as:<br>• CVD risk assessment and lifestyle education provided through a consultation with a physician and dietitian.<br>• Provision of information resources regarding lifestyle behaviour changes.<br>• Individualised 6-month lifestyle program through the Get Healthy Information & Coaching Service, which includes 10 counselling phone calls from a coach.

Abbreviations: CVD, cardiovascular disease; HDP, hypertensive disorders of pregnancy.

Table S2. Pearson's correlations between lifestyle behaviours and cardiometabolic outcomes at 6-months postpartum following HDP.

	BMI (kg/m2)	*Waist circum-ference (cm)*	*Fat mass %*	*Systolic BP (mmHg)*	*Diastolic BP (mmHg)*	*Number of children*
**Nutrition**						
Serves of vegetables/day	-0.072	-0.081	**-0.134***	-0.023	-0.008	-0.087
Above recommended 5 vegetables/day	-0.074	**-0.097***	-0.071	-0.059	-0.017	-0.05
Serves of fruit/day	**-0.189****	**-0.191****	-0.115	-0.034	-0.084	0.044
Above recommended 2 fruits/day	**-0.164****	**-0.146****	-0.085	-0.077	**-0.094***	-0.053
Above both 5 vegetables and 2 fruits/day	**-0.097***	**-0.113***	-0.054	**-0.125****	-0.078	-0.024
Processed meat serves/week	**0.136****	**0.120****	0.083	0.073	0.069	0.019
Serves of hot chips, French fries and wedges per week	**0.144****	**0.163****	0.086	0.082	0.075	-0.017
Serves of potato crisps or salty snacks/week	**0.119****	**0.149****	0.072	**0.151****	**0.122****	-0.001
Take-away occurrences/week	**0.133****	**0.147****	**0.138***	0.05	-0.001	0.056
Full fat milk	-0.001	0.059	-0.015	0.038	0.007	**0.100***
Cups of soft or sports drink/day	**0.277****	**0.281****	**0.232****	**0.114***	0.075	0.023
Cups of fruit juice/day	-0.027	0.029	0.028	-0.017	-0.008	-0.06
Cups of water/day	-0.021	-0.024	0.001	0.064	0.064	0.016
Alcoholic drinks/week	**-0.098***	-0.088	-0.012	-0.025	-0.038	**-0.108***
Alcohol intake occurrences/week	**-0.156****	**-0.142****	-0.034	-0.054	-0.049	**-0.105***
Consumed more than four alcoholic drinks in one intake in past 4 weeks	0.028	0.022	0.045	-0.046	0.025	0.042
**Physical activity**						
Mins of vigorous household chores in past week	0.065	**0.112***	**0.167****	**0.105***	0.04	**0.204****
Mins of vigorous yard work in past week	0.068	0.076	0.096	0.056	0.05	**0.103***
Mins of continuous walking in past week	**-0.131****	**-0.148****	-0.109	-0.035	-0.062	**-0.202****
Mins of vigorous PA in past week	**-0.093***	-0.09	-0.059	-0.06	-0.076	-0.054
Including activity previously mentioned, mins of strength/toning activity in past week	0.013	0.001	-0.108	-0.032	-0.043	-0.039
Combined mins of moderate-vigorous PA in past week◆	**-0.149****	**-0.161****	-0.115	-0.055	-0.084	**-0.190****
Meeting recommended 150minutes/week	**-0.167****	**-0.153****	**-0.117***	-0.083	**-0.108***	**-0.151****
Number of moderate-vigorous PA sessions in the past week◆	**-0.114***	**-0.103***	-0.098	-0.045	-0.075	**-0.127****
Meeting recommended 5 sessions/week	**-0.189****	**-0.193****	**-0.131***	-0.045	**-0.090***	**-0.173****
Meeting both recommended 150minutes in 5 sessions per week	**-0.196****	**-0.191****	**-0.150***	-0.081	**-0.124****	**-0.186****
Meeting recommendations for 150mins of PA in 5 sessions/week, as well as 5 vegetable and 2 fruit serves daily	**-0.117***	**-0.128****	-0.058	**-0.112***	**-0.104***	-0.054
**Other**						

Smoking status⊕	**0.112***	**0.142****	**0.131***	0.051	0.069	0.066
Average number of cigarettes smoked/day	0.708	0.608	0.438	0.032	0.311	-0.07
Electronic cigarette smoking status⊕	0.016	0.005	0.029	0.025	0.082	-0.015
Currently breastfeeding⊙	**-0.174****	**-0.210****	**-0.199****	**-0.103***	-0.07	-0.027
When stopped breastfeeding (months after birth)	-0.104	-0.132	**-0.240***	0.041	-0.02	-0.025

**Correlation is significant at the 0.01 level (2-tailed), bolded for convenience.

*Correlation is significant at the 0.05 level (2-tailed), bolded for convenience.

◆Includes minutes of moderate-vigorous physical activity and continuous walking per week.

⊙Categorical outcome recoded to demonstrate numerical direction; "0" for no, "1" for yes.

⊕Categorical outcome recoded in numerical order for magnitude; "0" for none or never, "1" for partial or previous, "2" for current.

Abbreviations: BMI, body mass index; BP, blood pressure; cm, centimetres; HDP, hypertensive disorders of pregnancy; kg, kilograms; mins, minutes; mmHg, millimetres of mercury; PA, physical activity.

Document S1. 6-month NSW Population Health Survey.

6M NSW Population Health Survey

Record ID

____

I have some questions to ask you about your general health, what you eat and drink including alcohol, your exercise and physical activity patterns, and cigarette smoking. Are you OK for me to go ahead and ask those questions now?
First I am going to ask a question about your general health.

Overall, how would you rate your health during the past 4 weeks?

- ◯ Excellent
- ◯ Very good
- ◯ Good
- ◯ Fair
- ◯ Poor
- ◯ Very poor
- ◯ Don't know
- ◯ Refused

Overall, how would you rate your mental health during the past 4 weeks?

- ◯ Excellent
- ◯ Very Good
- ◯ Good
- ◯ Fair
- ◯ Poor
- ◯ Very poor
- ◯ Don't know
- ◯ Refused

How do you feel about your life as a whole?

- ◯ Delightful
- ◯ Pleased
- ◯ Mostly satisfied
- ◯ Mixed
- ◯ Mostly dissatisfied
- ◯ Unhappy
- ◯ Terrible
- ◯ Don't know
- ◯ Refused

Now I would like to ask you some questions about alcohol.

How often do you usually drink alcohol?

- ◯ Record in days per week
- ◯ Record in days per month
- ◯ Less than once per month
- ◯ I don't drink alcohol
- ◯ Don't know
- ◯ Refused

How often do you usually drink alcohol in days per week?

____

How often do you usually drink alcohol in days per month.?

____

Alcoholic drinks are measured in terms of a "standard drink". A standard drink is equal to one middy of full-strength beer, one schooner of light beer, one small glass of wine or one pub-sized nip of spirits.

On a day when you drink alcohol, how many standard drinks do you usually have?

◯ Record number of drinks
◯ Don't know
◯ Refused

How many standard drinks per day?

____

In the past four weeks have you had more than two standard drinks in a day?

◯ Yes
◯ No
◯ Don't know
◯ Refused

In the past four weeks how often have you had seven or more drinks in a day?

◯ Record number of times
◯ Not at all
◯ Don't know
◯ Refused

In the past four weeks how many times have you had seven or more drinks in a day?

____

In the past four weeks how often have you had five-six drinks in a day?

◯ Record number of times
◯ Not at all
◯ Don't know
◯ Refused

In the past four weeks how many times have you had five-six drinks in a day?

____

In the past four weeks have you had more than four standard drinks on one occasion?

◯ Yes
◯ No
◯ Don't know
◯ Refused

How often do you have six or more standard drinks on one occasion?

◯ Daily or almost daily
◯ Weekly
◯ Monthly
◯ Less than monthly
◯ Never
◯ Don't know
◯ Refused

The next few questions are about food.

How many serves of vegetables do you usually eat each day? (One serve is half a cup cooked or one cup of salad vegetables).

◯ Answer in serves per day
◯ Answer in serves per week
◯ Don't eat vegetables
◯ Don't know
◯ Refused

How many serves of vegetables do you usually eat each day? (One serve is half a cup cooked or one cup of salad vegetables).

____

How many serves of vegetables do you usually eat each week? (One serve is half a cup cooked or one cup of salad vegetables).

____

How many serves of fruit do you usually eat each day? (A serve is one medium piece or two small pieces of fruit or one cup of diced pieces).

◯ Answer in serves per day
◯ Answer in serves per week
◯ Don't eat fruit
◯ Don't know
◯ Refused

How many serves of fruit do you usually eat each day? (A serve is one medium piece or two small pieces of fruit or one cup of diced pieces).

____

How many serves of fruit do you usually eat each week? (A serve is 1 medium piece or 2 small pieces of fruit or 1 cup of diced pieces).

____

How often do you eat processed meat products such as sausages, frankfurts, devon, salami, hamburgers, chicken nuggets, meat pies, bacon or ham?

◯ Answer in times per day
◯ Answer in times per week
◯ Answer in times per month
◯ Rarely or never
◯ Don't know
◯ Refused

How often do you eat processed meat products such as sausages, frankfurts, devon, salami, hamburgers, chicken nuggets, meat pies, bacon or ham per day?

____

How often do you eat processed meat products such as sausages, frankfurts, devon, salami, hamburgers, chicken nuggets, meat pies, bacon or ham per week?

____

How often do you eat processed meat products such as sausages, frankfurts, devon, salami, hamburgers, chicken nuggets, meat pies, bacon or ham per month?

____

How often do you eat hot chips, french-fries, wedges or fried potatoes?

◯ Answer in times per day
◯ Answer in times per week
◯ Answer in times per month
◯ Rarely or never
◯ Don't know
◯ Refused

How often do you eat hot chips, french-fries, wedges or fried potatoes per day?

____

How often do you eat hot chips, french-fries, wedges or fried potatoes per week?

____

How often do you eat hot chips, french-fries, wedges or fried potatoes per month?

____

How often do you eat potato crisps or other salty snacks such as twisties or corn chips?

◯ Answer in times per day
◯ Answer in times per week
◯ Answer in times per month
◯ Rarely or never
◯ Don't know
◯ Refused

How often do you eat potato crisps or other salty snacks such as twisties or corn chips per day?

____

How often do you eat potato crisps or other salty snacks such as twisties or corn chips per week?

____

How often do you eat potato crisps or other salty snacks such as twisties or corn chips per month?

____

What type of milk do you usually have?

◯ Regular milk (whole or full cream/dairy/soy/goats)
◯ Low /reduced fat milk (dairy/soy/goat)
◯ Skim milk (dairy/soy/goat)
◯ Evaporated or sweetened milk
◯ Other
◯ Don't have milk
◯ Almond/Rice/Oat milk
◯ Don't know
◯ Refused

Please specify 'other' milk

____

How many cups of soft drink, cordials or sports drink, such as lemonade or Gatorade, do you usually drink in a day? (one cup=250ml. One can of soft drink = 1.5 cups. One 500ml bottle of Gatorade = two cups).

◯ Cups per day
◯ Cups per week
◯ Doesn't drink soft drink
◯ Don't know
◯ Refused

How many cups of soft drink, cordials or sports drink, such as lemonade or Gatorade, do you usually drink in a day?

____

How many cups of soft drink, cordials or sports drink, such as lemonade or Gatorade, do you usually drink in a week?

____

How often do you have meals or snacks such as burgers, pizza, chicken or chips from places like McDonalds, Hungry Jacks, Pizza Hut, KFC, Red Rooster, or local take-away places?

◯ Times per week
◯ Times per month
◯ Rarely/Never
◯ Don't know
◯ Refused

How often do you have meals or snacks such as burgers, pizza, chicken or chips from places like McDonalds, Hungry Jacks, Pizza Hut, KFC, Red Rooster, or local take-away places in a week?

____

How often do you have meals or snacks such as burgers, pizza, chicken or chips from places like McDonalds, Hungry Jacks, Pizza Hut, KFC, Red Rooster, or local take-away places in a month?

____

How many cups of fruit juice do you usually drink in a day? (one cup=250ml, a household tea cup or large popper).

◯ Answer in cups per day
◯ Answer in cups per week
◯ Doesn't drink juice
◯ Don't know
◯ Refused

How many cups of fruit juice do you usually drink in a day?

____

How many cups of fruit juice do you usually drink in a week?

____

How many cups of water do you usually drink in a day? (one cup=250ml or a household tea cup).

◯ Number of cups per day
◯ Number of cups per week
◯ Doesn't drink water
◯ Don't know
◯ Refused

How many cups of water do you usually drink in a day?

____

How many cups of water do you usually drink in a week?

____

How much, if at all, does nutritional information influence the foods you purchase?

◯ Not at all
◯ A little
◯ A great deal
◯ Don't know
◯ Refused

Which items of nutritional information most influence your choice? (may select multiple responses)

☐ Protein
☐ Carbohydrates
☐ Fat
☐ Calories
☐ Sugar
☐ Salt
☐ Kilojoules
☐ Saturated fat
☐ Other (specify)
☐ Don't know
☐ Refused

Please specify 'other'

____

In your opinion how many kilojoules does the average person need in one day?

◯ 1, 200
◯ 4, 300
◯ 8, 700
◯ 16, 000
◯ Don't know
◯ Refused

Now I'm going to ask some questions about the physical activity you did in the LAST WEEK.

In the last week, how many times have you walked continuously for at least 10 minutes for recreation or exercise or to get to or from places?

____

What do you estimate was the total time you spent walking in this way in the last week (hours)?

____

What do you estimate was the total time you spent walking in this way in the last week (minutes)?

____

In the last week, how many times did you do any vigorous household chores which made you breathe harder or puff and pant? (do not include gardening)

____

What do you estimate was the total time you spent doing these vigorous household chores in the last week (hours)?

____

What do you estimate was the total time you spent doing these vigorous household chores in the last week (minutes)?

____

In the last week, how many times did you do any vigorous gardening or heavy work around the yard which made you breathe harder or puff and pant?

____

What do you estimate was the total time you spent doing vigorous gardening or heavy work around the yard in the last week (hours)?

____

What do you estimate was the total time you spent doing vigorous gardening or heavy work around the yard in the last week (minutes)?

____

In the last week, how many times did you do any vigorous physical activity which made you breathe harder or puff and pant? (eg: football, tennis, netball, squash, athletics, cycling, jogging, keep-fit exercises and vigorous swimming). Exclude house chores and gardening

____

What do you estimate was the total time you spent doing this vigorous physical activity in the last week (hours)?

____

What do you estimate was the total time you spent doing this vigorous activity in the last week (minutes)?

____

The next question does not include household chores or gardening. In the last week, how many times did you do any vigorous physical activity which made you breathe harder or puff and pant? (eg: lawn bowls, golf, tai chi, and sailing).

____

What do you estimate was the total time that you spent doing these activities in the last week (hours)?

____

What do you estimate was the total time that you spent doing these activities in the last week (minutes)?

____

Including any activities already mentioned, in the last week did you do any strength or toning activities? (Examples lifting weights, pull-ups, push-ups, or sit ups)

◯ Yes
◯ No
◯ Don't know/Not sure
◯ Refused

Including any activities already mentioned, in the last week how many times did you do any strength or toning activities? (Examples lifting weights, pull-ups, push-ups, or sit ups)

____

What do you estimate was the total time that you spent doing strength or toning activities in the last week (hours)?

____

What do you estimate was the total time that you spent doing strength or toning activities in the last week (minutes)?

____

The following questions are about tobacco smoking. This includes cigarettes, cigars and pipes. Please note that this does not include electronic cigarettes.

Which of the following best describes your smoking status?

◯ I smoke daily
◯ I smoke occasionally
◯ I don't smoke now but I used to
◯ I've tried it a few times but never smoked regularly
◯ I've never smoked
◯ Don't know

How many cigarettes per day do you smoke, on average? Is it....

◯ 1 to 10 cigarettes per day
◯ 11 to 20 cigarettes per day
◯ 21 or more cigarettes per day
◯ Don't know
◯ Refused

The last time you went to your GP, did the doctor discuss your smoking and advise you to quit smoking?

◯ Yes
◯ No
◯ Don't know
◯ Refused

Which of the following best describes how often you use electronic cigarettes?

◯ I've never used electronic cigarettes
◯ I've tried electronic cigarettes a few times but never used them regularly
◯ I don't use electronic cigarettes now, but I used to
◯ I use electronic cigarettes occasionally
◯ I use electronic cigarettes daily
◯ Don't know
◯ Refused

Ready to send surveys to the woman?

◯ Yes
◯ No
(Surveys will be emailed (five minutes after clicking the Yes button) to the email address listed in the 'Study Entry Contact Details' form)

Document S2. BP[2] study survey.

6M BP2 questionnaire

Many thanks for your commitment to the BP2 study. Please complete the surveys below. Please have your child's Health Record (Blue Book) handy before you start as you will need this to complete the surveys. Your hospital researcher will be in touch with you once you have completed the surveys to inform you of your group allocation.

Thank you

Lynne (BP2 Project Manager)

What ethnic group(s) do you identify with?

☐ Caucasian
☐ Asian
☐ Aboriginal or Torres Strait Islander
☐ Polynesian
☐ European
☐ Middle Eastern
☐ African
☐ Other

If other, please explain

____

What ethnic group(s) do your partner identify with?

☐ Caucasian
☐ Asian
☐ Aboriginal or Torres Strait Islander
☐ Polynesian
☐ European
☐ Middle Eastern
☐ African
☐ Other
☐ Not applicable

If other, please explain

____

What is your highest level of formal education?

◯ Secondary school
◯ Trade/Cert/Diploma
◯ University degree

What is your partner's highest level of formal education?

◯ Secondary school
◯ Trade/Cert/Diploma
◯ University degree
◯ Not applicable

What is/was your occupation? (before the baby was born)

____

What is/was your partner's occupation?

____

Current medications

Do you or anyone in your family have a history of the following? (When answering the family health questions, please only include first degree relatives - mother, father, brothers and sisters)

	You	Family
Stroke	☐	☐
Heart attack	☐	☐
Angina	☐	☐
High blood pressure	☐	☐
Kidney problems	☐	☐
Diabetes	☐	☐
Anxiety	☐	☐
Depression	☐	☐
Eating disorder	☐	☐
Significant illness	☐	☐
Other	☐	☐

If you answered yes to any of the above, please provide further details.

Did you have problems with falling pregnant?

◯ Yes
◯ No

If yes, please provide details

How many times have you been pregnant?

____

Of these pregnancies, how many have resulted in a miscarriage or termination?

____

Of these pregnancies, how many have resulted in a premature baby born before 37 weeks of pregnancy?

____

Of these pregnancies, how many have resulted in a full-term baby born after 37 weeks of pregnancy?

____

How many children do you have?

____

Are you currently pregnant?

◯ Yes
◯ No

If yes, how many weeks are you?

____

Are you currently in a relationship?

◯ Yes
◯ No

Is you current partner the father of the baby you had about 6 months ago?

◯ No
◯ Yes
◯ Not applicable

Have you had a change in partner between any of your pregnancies?

◯ No
◯ Yes
◯ Not applicable (first baby)

If yes, please provide details

Was the pregnancy that resulted in the birth of your baby 6 months ago, planned

◯ Yes
◯ No

Have you had high blood pressure in any of your pregnancies before the pregnancy with your current baby?

◯ Not applicable (first baby)
◯ No (I have had previous babies but no problems before this time with blood pressure)
◯ Yes

If yes, please provide details

Have you had gestational diabetes in any of your pregnancies?

◯ Yes
◯ No

If yes, please provide details

Are you currently breastfeeding?

◯ No (I didn't breastfeed)
◯ No (I have stopped now)
◯ Yes

When did you stop breast feeding?

Labour and birth experience

During labour and birth, I felt free to express my feelings

◯ Strongly disagree
◯ Moderately disagree
◯ Disagree
◯ Neutral
◯ Agree
◯ Moderately agree
◯ Strongly agree

During labour and birth, I felt I was

◯ Completely out of control
◯ Moderately out of control
◯ Out of control
◯ Neutral
◯ In control
◯ Moderately in control
◯ In complete control

Physically, I coped

◯ Worse than expected
◯ Moderately worse than expected
◯ Slightly worse than expected
◯ Neutral
◯ Slightly better than expected
◯ Moderately better than expected
◯ Better than expected

Emotionally, I coped

◯ Worse than expected
◯ Moderately worse than expected
◯ Slightly worse than expected
◯ Neutral
◯ Slightly better than expected
◯ Moderately better than expected
◯ Better than expected

When looking back at labour and birth, I feel

◯ Not at all proud of myself
◯ Moderately not proud of myself
◯ Slightly not proud of myself
◯ Neutral
◯ Slightly proud of myself
◯ Moderately proud of myself
◯ Very proud of myself

How would you describe your care during labour and birth?

◯ Very poor
◯ Moderately poor
◯ Slightly poor
◯ Neutral
◯ Slightly good
◯ Moderately good
◯ Very good

Please describe any things about your labour and/or the birth that you were particularly happy with

Please describe any things about your labour and/or the birth that you were particularly unhappy with

Since having your baby about 6 months ago,have you seen a doctor about your blood pressure?

◯ Yes
◯ No

If yes, please give details

Since having your baby about 6 months ago, have you seen your local doctor about any other health problems?

◯ Yes
◯ No

If yes, please give details

Since having your baby about 6 months ago, have you been referred to a specialist doctor for health problems?

◯ Yes
◯ No

If yes, please give details

Since having your baby about 6 months ago, have you seen your local doctor about any mental health concerns?

◯ Yes
◯ No

If yes, please give details

Since having your baby about 6 months ago, have you been referred to a specialist doctor or psychologist for mental health concerns?

◯ Yes
◯ No

If yes, please give details

Since having your baby about 6 months ago, have you been started on any new medications?

◯ Yes
◯ No

If yes, please give details

Have you experienced stress in the past 6 months (besides the stress of pregnancy and parenthood)?

◯ Yes
◯ No

If yes, was the stress due to?

☐ Financial difficulties
☐ Relationship worries
☐ Loss/death
☐ Housing changes
☐ Significant isolation
☐ Other

If other, please give details

Edinburgh Postnatal Depression Scale

Please select the answer that comes closest to how you have felt in the PAST 7 DAYS

I have been able to laugh and see the funny side of things

◯ As much as I always could
◯ Not quite so much now
◯ Definitely not so much now
◯ Not at all

I have looked forward with enjoyment to things

◯ As much as I ever did
◯ Rather less than I used to
◯ Definitely less than I used to
◯ Hardly at all

I have blamed myself unnecessarily when things went wrong

◯ Yes, most of the time
◯ Yes, some of the time
◯ Not very often
◯ No, never

I have been anxious or worried for no good reason

◯ No, not at all
◯ Hardly ever
◯ Yes, sometimes
◯ Yes, very often

I have felt scared or panicky for no very good reason

◯ Yes, quite a lot
◯ Yes, sometimes
◯ No, not much
◯ No, not at all

Things have been getting on top of me

◯ Yes, most of the time I haven't been able to cope at all
◯ Yes, sometimes I haven't been coping as well as usual
◯ No, most of the time I have coped quite well
◯ No, I have been coping as well as ever

I have been so unhappy that I have had difficulty sleeping

◯ Yes, most of the time
◯ Yes, sometimes
◯ Not very often
◯ No, not at all

I have felt sad or miserable

◯ Yes, most of the time
◯ Yes, quite often
◯ Not very often
◯ No, not at all

I have been so unhappy that I have been crying

◯ Yes, most of the time
◯ Yes, quite often
◯ Only occasionally
◯ No, never

The thought of harming myself has occurred to me

◯ Yes, quite often
◯ Sometimes
◯ Hardly ever
◯ Never

EPDS Score

____

General Anxiety Disorder Scale

Over the past 2 WEEKS how often have you been bothered by the following problems?

	Not at all	Several days	Over half the days	Nearly every day
Feeling nervous, anxious, or on edge	◯	◯	◯	◯

Not being able to stop or control worrying	◯	◯	◯	◯
Worrying too much about different things	◯	◯	◯	◯
Trouble relaxing	◯	◯	◯	◯
Being so restless that it's hard to sit still	◯	◯	◯	◯
Becoming easily annoyed or irritable	◯	◯	◯	◯
Feeling afraid as if something awful might happen	◯	◯	◯	◯

GAD-7 Score

____

If you checked off any problems, how difficult have these made it for you to do your work, take care of things at home, or get along with other people?

◯ Not difficult at all
◯ Somewhat difficult
◯ Very difficult
◯ Extremely difficult

Your Lifestyle

We would like to know what aspects of your lifestyle (like diet and exercise) are more or less satisfying to you. For each of the following questions, please check the box that best fits your situation.

	Not at all	Slightly	Somewhat	Quite a bit	Extremely
How satisfied are you with your weight when looking at it on the scales?	◯	◯	◯	◯	◯
How satisfied are you with the way you look?	◯	◯	◯	◯	◯
How satisfied are you with how much you eat?	◯	◯	◯	◯	◯
How satisfied are you with the taste of what you eat?	◯	◯	◯	◯	◯

Lifestyle 1

____

In seeking treatment for lifestyle change after hypertensive pregnancy, which of the following factors are important to you?

	Not at all important	Slightly important	Somewhat important	Quite a bit important	Extremely important
Being able to look good in your clothes	○	○	○	○	○
Reducing the risk or severity of high blood pressure, high cholesterol, diabetes, or heart disease	○	○	○	○	○
Having more energy	○	○	○	○	○
Living an active life	○	○	○	○	○
Feeling attractive	○	○	○	○	○

Lifestyle 2 ______________________________

Which of the following factors do you think you would find difficult to overcome when adopting a healthy lifestyle (such as diet or exercise)? Please check one answer per factor.

	Not at all difficult	Slightly difficult	Somewhat difficult	Quite a bit difficult	Extremely difficult
Cravings for unhealthy foods	○	○	○	○	○
Taste of unhealthy foods compared to healthy foods	○	○	○	○	○
Difficulty of preparing healthy meals	○	○	○	○	○
Difficulty of changing my eating habits because of my family	○	○	○	○	○
Difficulty of finding healthy meals when I go out to eat	○	○	○	○	○
Stress associated with sticking to any diet and exercise program	○	○	○	○	○
The need to eat when I am frustrated, stressed, anxious, or sad	○	○	○	○	○
Fitting diet and exercise in my schedule	○	○	○	○	○
Fitting diet and exercise in my life because of my other health conditions	○	○	○	○	○

Lifestyle 3 ______________________________

Optional interview - PLEASE NOTE THAT THIS PART OF THE BP2 STUDY IS NOW FINISHED

The research team is keen to hear about your experiences of looking after your own health after having a baby and your views of this program. Would you be interested in participating in a phone interview later this year, when your baby is approximately 10 months old? This interview is entirely voluntary. It would take about 45-60 minutes at a time that suits you.

◯ Yes
◯ No

Thank you for your offer of participating in an interview. THIS PART OF THE BP2 STUDY IS NOW FINISHED and we no longer need women to volunteer for an interview.

References

1. Henry, A., et al., *Blood pressure postpartum (BP2) RCT protocol: Follow-up and lifestyle behaviour change strategies in the first 12 months after hypertensive pregnancy.* Pregnancy Hypertens, 2020. **22**: p. 1-6.
